# Perioperative oncolytic virotherapy to counteract surgery-induced immunosuppression and improve outcomes in pancreatic ductal adenocarcinoma

**DOI:** 10.3389/fonc.2023.1071751

**Published:** 2023-02-16

**Authors:** Sarah Mansouri, Lauren Daniel, Nawal Amhis, Maxime Leveille, Jeanette E. Boudreau, Almohanad A. Alkayyal, Yves Collin, Lee-Hwa Tai

**Affiliations:** ^1^ Department of Immunology and Cell Biology, Université de Sherbrooke, Sherbrooke, QC, Canada; ^2^ Department of Surgery, Université de Sherbrooke, Sherbrooke, QC, Canada; ^3^ Department of Microbiology and Immunology, Dalhousie University, Halifax, NS, Canada; ^4^ Department of Medical Laboratory Technology, Faculty of Applied Medical Sciences, University of Tabuk, Tabuk, Saudi Arabia; ^5^ Immunology Research Program, King Abdullah International Medical Research Center, Riyadh, Saudi Arabia; ^6^ Research Center of the Centre hospitalier universitaire de Sherbrooke (CHUS), Sherbrooke, QC, Canada

**Keywords:** pancreatic ductal adenocarcinoma (PADC), surgery, perioperative period, neoadjuvant, oncolytic virus, tumor microenvironment, immunosuppression, pancreatic cancer

## Abstract

Pancreatic ductal adenocarcinoma (PDAC) is a high fatality cancer with one of the worst prognoses in solid tumors. Most patients present with late stage, metastatic disease and are not eligible for potentially curative surgery. Despite complete resection, the majority of surgical patients will recur within the first two years following surgery. Postoperative immunosuppression has been described in different digestive cancers. While the underlying mechanism is not fully understood, there is compelling evidence to link surgery with disease progression and cancer metastasis in the postoperative period. However, the idea of surgery-induced immunosuppression as a facilitator of recurrence and metastatic spread has not been explored in the context of pancreatic cancer. By surveying the existing literature on surgical stress in mostly digestive cancers, we propose a novel practice-changing paradigm: alleviate surgery-induced immunosuppression and improve oncological outcome in PDAC surgical patients by administering oncolytic virotherapy in the perioperative period.

## 1 Background

Pancreatic ductal adenocarcinoma (PDAC) is a leading cause of cancer-related morbidity and mortality in the western world. A recent study by Rahib et al. demonstrated the rising global incidence of PDAC that is expected to surpass colorectal cancer to become the second leading cause of cancer-related deaths by 2030 (1). Whereas survival in various solid tumors has constantly improved in the last decade, only mild advances have been achieved in PDAC. A 5-year overall survival of only 11% (2), insufficient/limited treatment options and rising incidence, warrant the development of new therapeutic strategies against PDAC.

Metastatic disease is a main contributor to poor outcomes in PDAC (3, 4). By the time patients are diagnosed with pancreatic cancer, most cases are at a locally advanced or metastatic stage (5). This is in part attributed to the lack of symptoms and appropriate screening tools resulting in 80% of patients presenting with metastatic disease at initial diagnosis (5). For these patients, the 5-year overall survival drops drastically to less than 1% as there are no curative treatments and survival is measured in months (6). Currently, surgery remains the only curative treatment for PDAC. However, less than 10 to 20% of patients newly diagnosed with PDAC are eligible for surgery (7). Patients who can receive surgical care have the best prognosis, with a 5-year overall survival of 20% following surgery (5). Despite the curative intent of surgical resection, over 60% of patients will recur within the first two years after surgery (8, 9). This further supports the pressing need to develop new therapies and understand the mechanisms behind recurrence and metastatic spread of pancreatic cancer.

The paradoxical phenomenon of recurrence and metastatic spread following surgical resection has been documented in many solid cancers (10–12). While the underlying mechanism is not fully understood, there is compelling evidence to think surgery could play a role in residual disease progression, specifically in PDAC. It has been described that surgery creates a state of physical trauma and physiological stress that triggers cellular immune dysfunction (13, 14). Our group and others have shown that the postoperative period represents a unique time frame of immunosuppression that can be hijacked by a tumor for its survival advantage in different cancer types (15–18). However, the idea of surgery-induced immunosuppression as a facilitator of recurrence and metastatic spread has not been explored in the context of pancreatic cancer.

## 2 Standard of care for PDAC

Currently, surgery offers the only realistic chance to cure pancreatic cancer. However, less than 10 to 20% of patients newly diagnosed with PDAC are resectable (7, 19). Patients with tumors located in the head or the uncinate process of the pancreas are offered a pancreatoduodenectomy (PD-Whipple procedure), whereas tumors of the body or tail of pancreas are removed by performing a distal pancreatectomy and splenectomy (5). These procedures are amongst the most complex and aggressive cancer surgeries. Despite tremendous progress in patient care, patients present with long recovery time and high complication rates. Post-operative complications range from wound infection to catastrophic pancreatic fistula and even death (4, 5). Yet, 5-year overall survival after surgical resection alone remains poor at 10% (20, 21). Therefore, 6 months of adjuvant chemotherapy is combined with resectable PDAC because it improves long term survival after surgery (22). Patients with high functional status are offered a postoperative chemotherapy regimen composed of FOLFIRINOX, a highly effective, but cytotoxic chemotherapy. Patients with low functional status, on the other hand, are assigned gemcitabine and capecitabine based chemotherapy, a less effective, but more tolerable agent (22). However, despite adjuvant therapy, recurrence rates remain high, with 69 to 75% of patients experiencing relapse within 2 years following their surgery (4).

Patients with metastatic disease (locally advanced-20-40% and metastatic disease-40-60%) have a far grimmer prognosis. Survival at 5 years is well below 5% (19). The treatment offered is palliative chemotherapy (23). In fact, gemcitabine has been the first line of treatment of metastatic disease for the last 14 years since research has shown its therapeutic advantages in extending progression-free survival (PFS). Regrettably, even with gemcitabine, the overall survival (OS) of patients with metastatic illness is between five and six months (19, 23). The goal of therapy is to control disease progression and symptoms with quality of life as endpoint.

In the last decade, immunotherapy, and more specifically immune checkpoint inhibitors have emerged as a promising therapeutic avenue for PDAC. This modality relies on infiltration of the tumor by the immune system to induce a meaningful antitumor effect (24). However, PDAC has been shown to mostly resist immunotherapy (25–28). This can be attributed to the immunosuppressive tumor immune microenvironment (TIME) and scarce infiltration by effector T cells (29, 30). A small sub-population of PDAC patients respond to anti-PD1 checkpoint inhibitors. These patients have tested positive for specific gene changes, such as a high level of microsatellite instability-high (MSI-H) or changes in the mismatch repair (MMR) genes, which has led to the inclusion of anti-PD1 as second line treatment in recurrent and metastatic disease (23, 31, 32). However, less than 1% of PDAC patients express this genetic signature to benefit from the use of anti-PD1 (3).

## 3 Surgery induced immunosuppression in solid cancers

**Figure 1:**
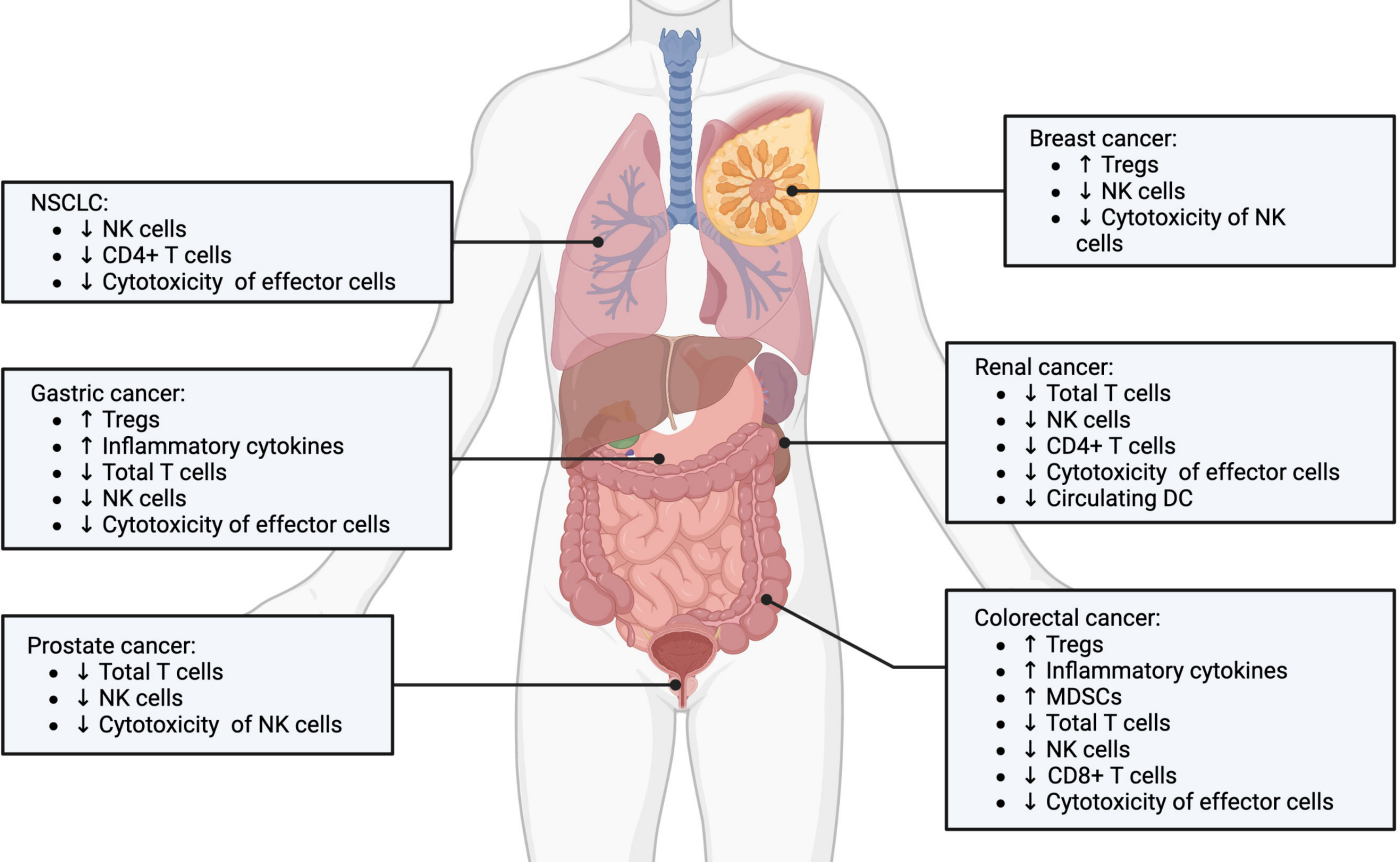
Postoperative immunosuppression in various solid cancer types. Postoperative immunosuppression is a stress and inflammation induced response that is observed across many types of solid tumors, including non-small cell lung, gastric, prostate, breast, renal and colorectal cancers. The underlying cellular mediators involve expansion of pro-tumorogenic regulatory immune cells and dysregulation of tumor-targeted effector immune cells. Created with BioRender.com.

Immune suppression following surgical resection is a now well-established concept. This phenomenon can be observed across all types of solid tumors (**Figure 1**). For example, following surgery for colorectal cancer, interferon production was inhibited and NK cell function profoundly impaired (33). In addition, surgery reduced the overall amount of CD8+T cells, increased the number of myeloid derived suppressor cells (MDSC) in the peritoneal cavity, which was associated with worst overall survival for patients (34). Similarly, postoperative immunosuppression has also been demonstrated in lung (35–38), renal (39, 40), gastric, breast (41, 42), and prostate cancer (43). Systematically, the depth of immune suppression was directly correlated to the extent of surgical resection across these cancer types.

The physical act of surgical excision instigates a stress response that provokes a surge in cortisol and catecholamines by the sympathetic nervous system (SNS), leading to the activation of physiological mechanisms such as the inflammatory response and angiogenesis that aids in tissue and wound healing. However, angiogenesis and inflammation have also been linked to the survival of residual tumor cells, metastasis, and recurrence of cancer (44, 45). Surgery related risk factors, including duration of surgery, extent of tissue damage, blood loss, hypothermia, and the administration of blood products have all been shown to contribute towards postoperative immunosuppression (14, 33, 45–49). These mediators of stress in turn raise levels of immunosuppressive molecules, such as IL-4, IL-10, TGF-β, and VEGF; and pro-inflammatory cytokines, such as IL-6 and IL-8 (50). These cytokine alterations have been found systemically and in longitudinal tumor resections following surgery (50). Previous and current studies connect these altered cytokine levels with suppression of the effector functions of tumor-targeted NK and effector T cells in the postoperative period (15, 16, 51–54). The activated SNS also promotes the growth of Th2 cells and T regulatory cells (Treg), consistent with Treg’s known functions in inflammation control and tissue healing (17, 45, 50, 55). Surgery induced immunosuppression is further reinforced through the expansion of MDSCs (56–58). These myeloid regulatory cells have been shown to promote tumor growth in the postoperative period by regulating the development of premetastatic niches, stimulating angiogenesis, and promoting tumor cell invasion (16, 45, 59). Notably, cancer prognosis and recurrence in patients with solid cancers are correlated with postoperative MDSC expansion and suppressive function (45). Postoperative cellular immunosuppression has been demonstrated to last several weeks following surgery, with overall dysfunction in NK and T cell function (16, 60) and decreased total lymphocyte counts (61). Studies have described a return to normal levels of cellular immunity weeks to a month after surgery (61, 62). However, recent clinical data suggest that cellular immunity may be compromised for up to 6 months following surgery (63).

Of note, neutrophils have been demonstrated to serve a critical role in tissue repair following surgical injury. This is in addition to their well-documented functions in defending against infection, and both promoting and guarding tumor growth and spread (64). In different cancer models, tumor-associated neutrophils (TANs) are linked with disease progression and poor overall survival (65–67). In the context of cancer surgery, neutrophils have been described to release neutrophil extracellular traps (NETs) that attract cancer cells. Surgical procedures induce the production of NETs, which can encourage the development of metastases by shielding against anti-tumorogenic factors and tumor-targeted immune cells (67, 68)

**Figure 2:**
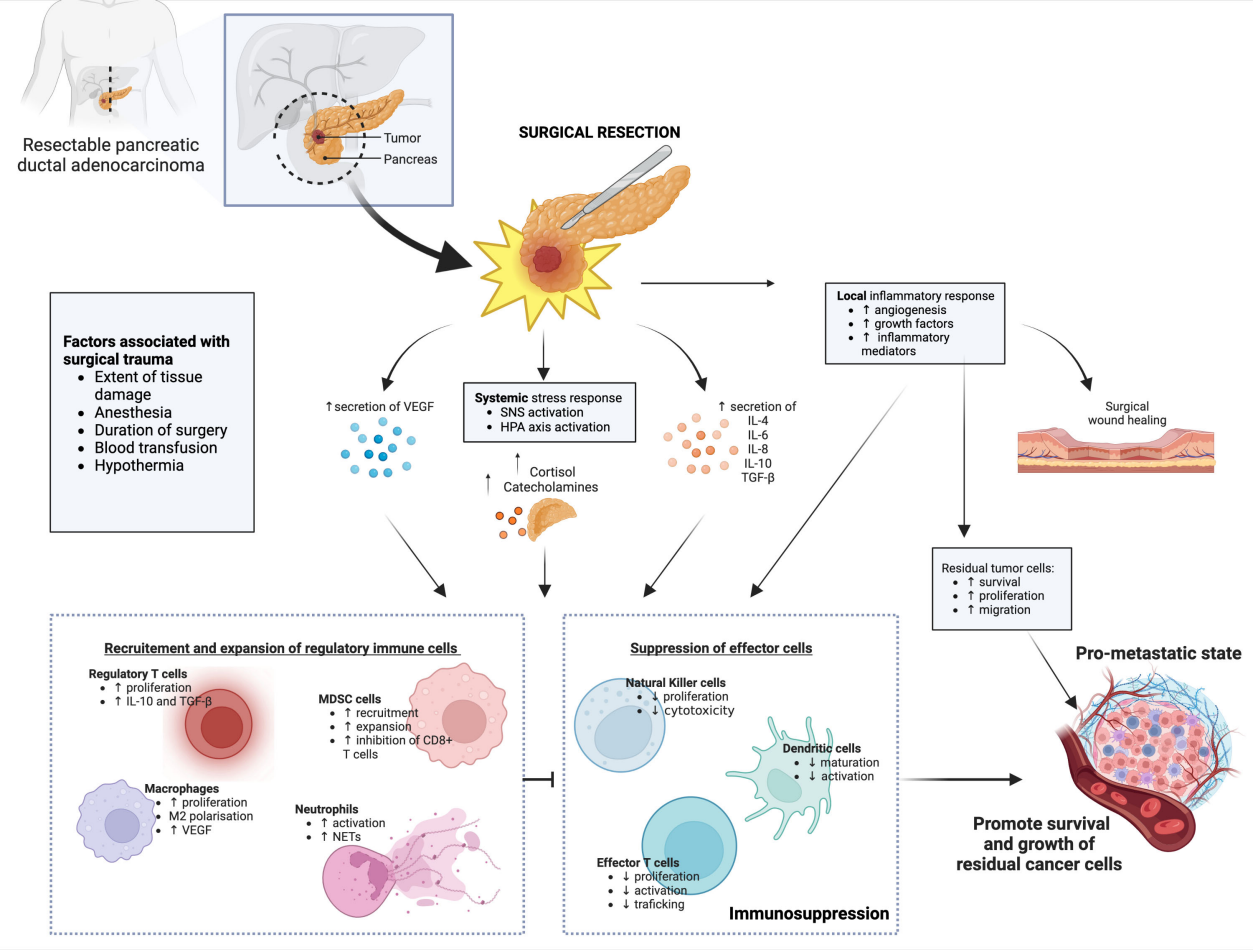
Schematic representation of the mechanisms of postoperative immunosuppression. Surgical resection of pancreatic cancers initiates local and systemic inflammatory and stress responses, which leads to dysregulated cytokine, growth factors and stress hormone secretion levels. While necessary for tissue and cellular repair following surgical excision, these inflammatory responses result in expansion and recruitment of regulatory immune cells, which in turn impair tumor-targeted effector immune cells. When combined with other negative factors associated with surgical trauma, such as duration of anesthesia, blood loss and hypothermia - a pro-metastatic state establishes in the patient, which promotes growth and migration and residual cancer cells. Abbreviations: SNS, Sympathetic nervous system; HPA, hypothalamic-pituitary-adrenal; VEGF, vascular endothelial growth factor; TGF-β, Transforming growth factor beta; MDSC, myeloid derived suppressor cells; NET, neutrophil extracellular traps; IL, interleukins. Created with BioRender.com.

Taken together, these studies suggest the complex interplay between different immune cell populations, soluble mediators and cancer cells that favor the creation of a local and systemic immunosuppressive tumor microenvironment (TME) (**Figure 2**). This in turn tilts the balance in favor of promoting the growth of residual tumor cells, which is a critical factor in the capacity of tumors to evade both innate and adaptive immunity.

## 4 Surgery induced immunosuppression in pancreatic cancer

Although we have previously demonstrated that there is a clear correlation between surgery and immune suppression in different solid tumors, there is a dearth of literature in the context of surgical resection for PDAC. Romano et al. was one of the first to show that compared to other gastrointestinal resectable tumors, PDAC surgical patients exhibited higher immune suppression in the postoperative period. There was a statistically significant reduction in total lymphocyte counts (TLC) at days 14 and 50 postoperative, comparing pancreatic to gastric/colorectal patients (69). Many other reports corroborate this finding of altered patient immune status following pancreatic resection leading to poorer survival (8, 70, 71). D’Engremont et al. demonstrated post-operative lymphopenia was an independent risk factor of mortality and lower survival in localized pancreatic cancer in a risk prediction model of 390 resectable PDAC patients. Four years after surgery, only 4.6% of patients with post-operative low TLC (under 1000/mm^3^) were alive compared to 22.8% of patients with a post-operative high TLC (above 1000/mm^3^) (70). The aggressive nature of PDAC was used to explain these outcomes without consideration of potential underlying immunosuppressive mechanisms leading to poorer oncologic outcomes.

More recently, Kim et al. confirmed that PDAC patients who successfully underwent curative pancreatectomy, exhibited changes in their immune status following surgery. In this study, most patients had normal TLC levels preoperatively. However, following surgery, TLC levels fell dramatically and neutrophil to lymphocyte ratios (NLR) spiked for 3 to 4 days after. These counts generally increased after the initial postoperative period but could remain altered for as long as 6 months. They showed that low TLC and high NLR in the post-operative period was an important predictor of poor outcome, while both led to worst overall and disease-free survival at 1, 2 and 3 years. When stratifying for NLR, a high value at 1 and/or 6 months postoperatively was a significant risk factor for PDAC recurrence (61). In a separate study by Pointer et al., a high NLR was predictive of patient survival and associated with poor prognosis after resection in early-stage PDAC (62). Postoperative neutrophil recruitment and NET formation, through the activation of STAT3 and NFκB pathways have been associated with recurrence and metastatic progression. This data could provide some mechanistic underpinnings to explain the above clinical observations (11, 72).

Our group previously demonstrated that surgical trauma induces profound NK cell dysfunction leading to poorer outcomes in preclinical models. We hypothesized that poor prognosis was mainly driven by NK cells which we know to play a crucial role in cancer cell clearance and immunosurveillance in humans (15, 57, 73). In 2015, Iannone et al. demonstrated that pancreatic cancer surgery decreased NK cell numbers and impaired their function, particularly their ability to release IFN-γ (74). Through a comprehensive examination of peripheral lymphocyte subsets in pancreatic cancer patients before and at various intervals after duodenopancreatectomy, they were able to demonstrate an alteration in NK cell numbers and function in patients at post-operative days 7 and 30. The same authors also performed an observational analysis performed on the small subset of patients who survived at 2-years. Their findings suggest the importance of NK cell frequency. In fact, survivors (8/12 patients) exhibited a median NK cell frequency distribution at post-operative day 30 that was significantly higher than that of deceased patients; this holds true even when advanced cancer patients were considered. At two months after surgery, NK cell numbers and function normalized for all patients. The authors suggest that the dysregulation of NK cell–modulating cytokines, such as IL-2, IL-12, and IL-18, an CD4/CD25 regulatory T-cell subsets in pancreatic cancer patients could set up an altered microenvironment, in which NK cells adopted aberrant proliferation, differentiation, and functional behavior, which correlated with patient survival.

While we await more mechanistic studies, clinical evidence provides support for the altered antitumor host defense after pancreatic resection and its effect on cancer outcome. Many groups refer to the unique period following surgery as a “perioperative window of opportunity” that if acted on could potentially alter the course of disease prognosis (45, 47, 49, 75). However, in a cancer where surgery is the only curative option, it is paramount to have a better understanding of the PDAC TIME, in order to turn this postoperative immunosuppression into a therapeutic window of opportunity.

## 5 The tumor microenvironment in PDAC

The TIME is classified according to the degree of immune T cell infiltration. Immunologically “hot” tumors display high rates of effector T cell infiltration. Classically “hot” cancers include melanoma, bladder, kidney, head and neck, and non-small cell lung cancer. On the other hand, “cold” tumors are nonimmunogenic as they exhibit low rates of effector T cell infiltration. Ovarian, prostate, brain and pancreatic cancers are typically cold tumors (24). The TIME has been shown to be a critical determinant of immunotherapy resistance. PDAC is one of the most stroma dense tumors, with up to 90% of its tumor volume made up of extracellular matrix (ECM) (76). This highly dense and fibrous stroma contributes to increased intra-tumoral pressure. The scar tissue-like desmoplasic stroma creates a physical barrier around the tumor cells, while the hypoxic gradients recruit fibroblasts and leukocytes, which together impedes both the entry and efficacy of existing therapeutics (77, 78). In response to factors secreted by the tumor and the hypoxic microenvironment, cancer-associated fibroblasts (CAF) secrete high quantities of collagen and extra-cellular matrix, contributing further to the desmoplasia. Moreover, their cross talk with multiple components of the immune system helps to maintain a stubborn state of immune suppression (79, 80).

As for immune infiltrates, their presence in PDAC is complex and heterogeneous. Myeloid cells are the most prominent population (>50%) and are associated with worse prognosis in patients with surgically resected PDAC. MDSCs are present early in the carcinogenesis of PDAC and play a central role in the immunosuppressive environment (81–83). Likewise, tumor associated macrophages (TAM) are in relative abundance in PDAC. Macrophages are polarized to their immunosuppressive M2 phenotype and are thought to increase in number with invasiveness of the tumor and are associated with worse clinical outcomes (55). In terms of dendritic cells (DCs), they are present in low numbers and are mostly immature, rendering them dysfunctional (84). NK cells, which are essential for the recognition and elimination of cancer cells, are not frequent and are mostly dysfunctional (81). Similarly, PDAC tumors present few tumor infiltrating lymphocytes (TILs) and most of them are Treg cells, a subset of T-cells known for their immunosuppressive activity. As for effector CD8+ cytotoxic T-cells and CD4+ helper T-cells, they are found in lower overall proportion in PDAC. Regarding their spatial contexture, tumor infiltrating CD3+ T-cells are located far from cancer cells and are trapped within the dense stroma. Furthermore, these CD3+ T cells are most often found in a state of exhaustion and less than 1% demonstrate tumor reactivity (30). It is well established that the presence of TILs and DCs is associated with better prognosis for patients. Overall, this paints a heavily immune suppressed TIME, which favors tumor escape and therapy resistance (28, 55).

## 6 Immune evasive mechanisms in PDAC

The immune evasion mechanisms employed by PDAC allows the cancer to proliferate and spread months to years before the first clinical symptoms are detected (85). This can be partially attributed to pancreatic tumor cell-intrinsic pathways, driven by mutated KRAS. Oncogenic mutated KRAS is found in up to 90% of patients and is thought to be the driving mutation in PDAC oncogenesis (4). Pro-tumorogenic cytokines and chemokines, such as IL-6, IL-8, IL-10, TGF-β, M-CSF, VEGF and CXCL12, are released by PDAC cells and are tightly controlled by oncogenic KRAS dependent mechanisms. The abundant release of these soluble mediators tips the immunological balance from effective immune surveillance to immune escape. The proliferation, migration, and angiogenesis of tumor cells are all influenced by these immunological alterations (86). The secretion of this cocktail of modulators recruits and activates immunosuppressive cells, such as MDSCs, TAMs, Treg cells, CAFs, Th2 cells, and neutrophils into the TIME (87). The recruitment of these immunosuppressive cell subsets coupled to the heavy release of pro-tumorogenic cytokines listed above prevent the anti-tumor functions of NK and CD4+/CD8+ T cells and prevent the maturation and survival of DCs (82, 86). Since DCs are potent antigen-presenting cells (APCs) that are crucial for activating an effective anti-tumor T cell response, lower DC levels in blood and tumor tissue of PDAC patients have been correlated with diminished survival (56). PDAC also possesses a low mutation burden, another feature which allows it to evade the immune system (88). The resulting absence/rarity of neoantigens, which act as immune targeting molecules necessary to induce a productive T cell response, also contribute to poor infiltration by T cells (89).

In parallel, PDAC cells downregulate their antigen presentation machinery. This is achieved by the downregulation of major histocompatibility complex (MHC) class I molecules, which allows cancer cells to escape CD8+ T cell recognition (90). Furthermore, there is an upregulation of inhibitory immune checkpoint ligands, including PD-L1, which promotes T-cell dysregulation and loss of effector function (91).

Through the activation of CAFs, PDAC is also able to physically restrict anti-tumor immune cells from entering the TME, in addition to immunological evasion (92, 93). Through collagen deposition and ECM reorganizations, these CAFs encourage fibrosis, which causes a desmoplastic reaction. This creates a physical barrier that prevents normal vascularization, hinders the infiltration of anti-tumor immune cells, and contributes to therapeutic resistance (79, 92, 94). The dense fibrous stroma of PDAC further contributes to immune evasion by creating a hypoxic and hostile environment for effector T cells.

VEGF overexpression is a frequent finding in biopsies of human pancreatic tumors, demonstrating the importance of hypoxia in PDAC pathophysiology (95, 96). Hypoxia is thought to influence tumor development dynamics and confers immunotherapeutic resistance. Hypoxia-induced production of VEGF, along with other cytokines (such as IL-10, IL-6, and G-CSF) inhibit DC maturation, hence decreasing overall numbers of DCs and rendering dysfunction in CD3+ T cells. In addition, matrix metalloprotease type-9 (MMP-9) generated by tumor-associated neutrophils (TANs) and macrophages in the TIME potentiate the effects of VEGF. Indeed, the overexpression of MMP-9 seen in PDAC plays a critical and complex role in immune suppression, tumor progression, invasion, and metastasis (94, 97). During carcinogenesis, MMP-9 increases endothelial cell migration and activates the angiogenic switch in tumors through the release of VEGF from the matrix, suggesting the pro-angiogenic role of MMP-9 in cancer tissues (98). In contrast, the direct proteolytic action of MMP-9 leads to cancer spread, most likely *via* the control of VEGF and angiostatin production and in association with angiogenesis (99, 100). This suggests that the increased vascular permeability leading to early dissemination of PDAC is partly induced by the over-expression of MMP-9 in the TME (98, 101).

Hypoxia also plays a role in immune evasion through the activation of pancreatic stellate cells (PSC). PSC are myofibroblast-like cells that regulate the synthesis of extracellular matrix (ECM). Although the synthesis, deposition, and remodeling of fibrous connective tissue is protective at steady state, over-activated PSCs hinder normal pancreatic function. Recently, PSCs have emerged as a focal point in pancreatic cancer research in their potential regulation of PDAC carcinogenesis. There are emerging data describing their contributing towards the depletion and malfunction of T cells as well as chemoresistance (21). Activated PSCs govern T-cell migration and block anti-tumor CD8+ T-cells from approaching cancer cell clusters, thus reducing cancer cell access (102, 103). Activated PSCs also accelerate the polarization of M1 macrophages towards a pro-tumoral M2 phenotype (104).

## 7 Treating postoperative immunosuppression in PDAC with oncolytic virotherapy

Very little research has been conducted on overcoming surgery induced immunosuppression in PDAC using immunomodulators aside from a few studies of perioperative cytokine and chemotherapy delivery. In 2008, a clinical trial was conducted to determine the effect of presurgical IL-2 immunotherapy on immune status and survival in PDAC patients undergoing pancreatic resection. Thirty consecutive patients undergoing a Whipple (pancreatoduodenectomy) procedure were randomly assigned preoperative IL-2 immunotherapy plus surgery or surgery alone. In the immediate post-operative period, patients treated with IL-2 displayed no post-operative lymphopenia as they had higher total lymphocyte counts compared to the control group. After 36 months of follow-up, the free-from-progression period (FFPP) and OS were increased in patients treated with IL-2 (105). This underpowered study provided evidence that immune modulation in the perioperative period could lead to improved oncological outcomes. However, the low half-life and potential toxicity of systemic IL-2 therapy limited its clinical progress.

**Figure 3:**
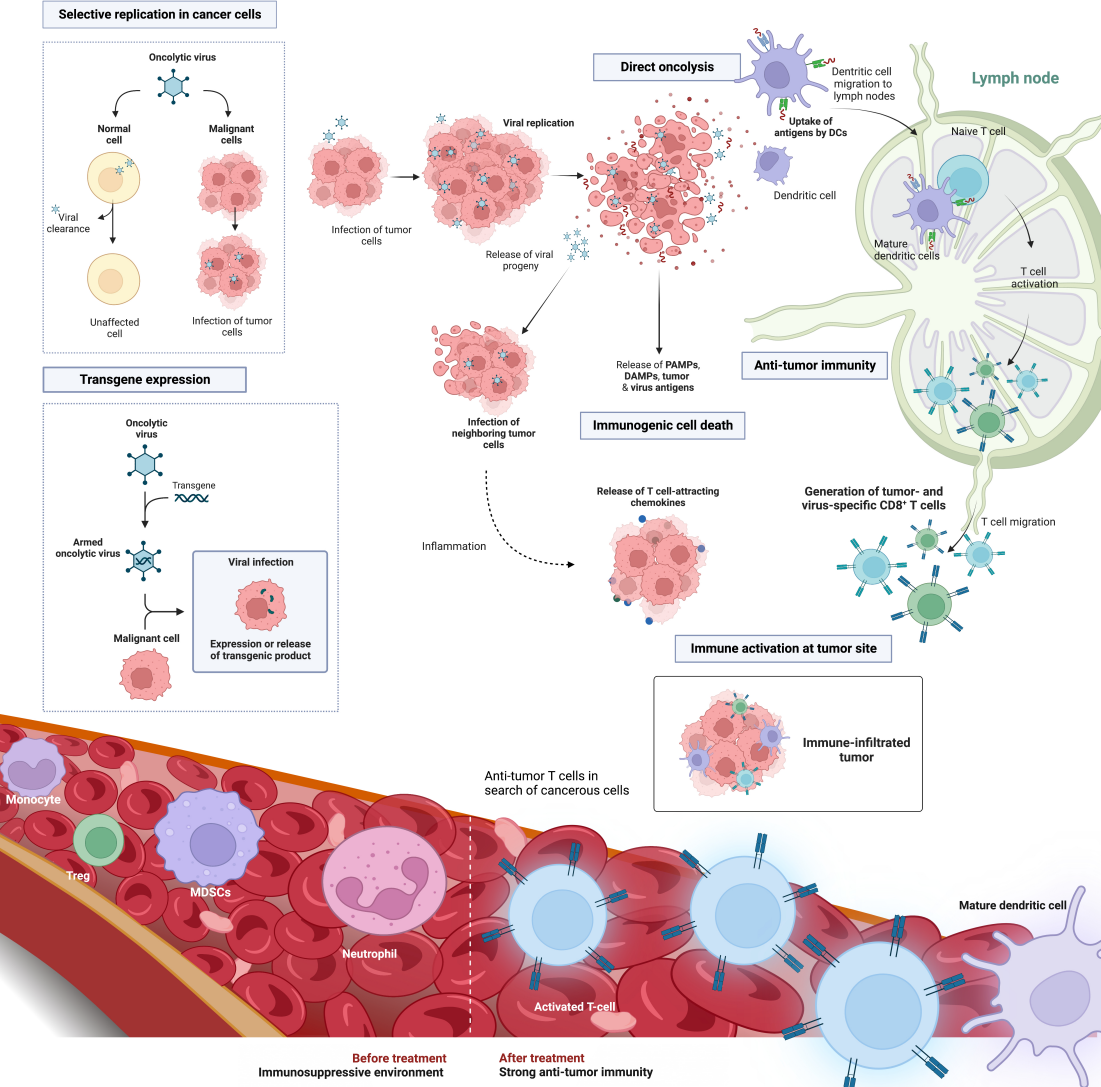
Oncolytic virus platform in the treatment of solid cancers. OVs are a versatile tumor targeting therapeutic platform involving direct tumor cell oncolysis, immune activation and transgene expression to enhance tumor lysis. Selective oncolysis enables viral replication as well as infection and lysis of both neighboring and distant tumor cells. Viral replication results in the release of both viral and tumor-associated antigens. Depending on the viral backbone, oncolysis is characterized by an immunogenic form of cell death and the release of immunomodulatory molecules such as DAMPs and PAMPs. This causes the recruitment and maturation of dendritic cells that may cross-present TAAs to CD8+T cells, hence producing TAA-specific T-cell populations. These biomarkers of ICD recruit and activate tumor-targeted immune T cells that infiltrate the TME. OVs can also be engineered to harbor select transgenes that can further enhance tumor eradiation. Examples include immune stimulating cytokines, tumor-targeted miRNAs, and reporter genes to track viral infection. Abbreviations: OV, oncolytic virus; ICD, immunogenic cell death; DAMPs, danger associated molecular patterns; PAMPs, pathogen associated molecular patterns; TAA, Tumor associated antigen; TME, tumor microenvironment; miRNA, microRNAs. Created with BioRender.com.

Compared to other immunotherapeutic modalities, oncolytic viruses (OV) attack cancer cells in a multimodal way. OVs are a class of naturally occurring or bioengineered live therapeutics that selectively infect, replicate within, and lyse cancer cells, resulting in immunogenic cell death (ICD) of the tumor cells (106). The activation of cell stress and danger pathways along with the release of otherwise hidden tumor associated antigens (TAA), recruits and activates tumor-targeted immune effector cells. The tumor is prevented from eluding the immune system and results in a rapid, specific, and long-lasting anti-tumor immune response against TAA, both at the primary tumor site and at distant metastatic sites (106, 107) (**Figure 3**). While the use of OV for PDAC treatment has been explored in numerous preclinical and clinical settings (108–114), the timing of OV administration in the surgical context nor its potential to combat postoperative immunosuppression have been investigated in the setting of PDAC. Increasing preclinical and clinical evidence supports the benefits of neoadjuvant systemic therapy for all patients with resectable PDAC (20, 115). Kubo et al. examined the association between neoadjuvant therapy and pathological outcomes in patients with PDAC following resection. They found that patients who received neoadjuvant therapy had lower rates of micro-vascular invasion (MVI) (43%) compared to patients who had surgery without neoadjuvant treatment (62%). Patients with MVI had an odds ratio of 2.38 to experience liver recurrence and MVI was found to be an important prognostic factor (116). These findings highlight the importance of the neoadjuvant treatment period and reinforces the importance of research to rationalize timing and combination treatments to optimize outcome for resectable PDAC patients. When it comes to OV dosing and administration strategy, Warner et al. summarize it into 3 categories: (1) Hit hard and early – the administration of a highly concentrated dose once and early in the treatment regimen; (2) kill softly - frequent small doses until tumor regression; and (3) prime-boost approach – high dose of a first virus to prime an immune response, followed days to weeks later by a second high dose of a different virus to boost the anti-tumoral immune response and maximize oncolysis without the rapid antigen-based viral clearance targeted at the prime virus (117). For now, there is no clear evidence to which strategy is best when it comes to PDAC. Given that surgery is the only chance for cure in PDAC patients, we postulate that perioperative oncolytic virotherapy administered to PDAC patients immediately before or after surgery may prime anti-tumor immune responses and prevent or reduce postoperative immunosuppression. Cell-mediated immunity may be more effectively activated immediately prior to the immunosuppressive effects of surgery (49, 118). Our group previously showed in a mouse model of surgical stress that neoadjuvant administration of oncolytic vaccinia virus can reverse NK cell dysfunction and reduce postoperative colorectal lung metastases (15, 53, 57). In a phase 1b window-of-opportunity trial, 9 patients bearing colorectal liver metastases or metastatic melanoma scheduled for surgery, went on to receive neoadjuvant oncolytic vaccinia virus (JX-594) 10 to 22 days prior to surgery (119). Neoadjuvant OV increased both NK and T cell functional anti-tumor immune responses in all patients. Moreover, activated T cell response remained for up to 3 months following viral infusion in all patients. Notably, these functional immune findings translated to increased survival. At the 3-year follow-up, 5 of 9 patients were still alive and 3 patients remained cancer-free. While these results were not obtained from PDAC patients, it is not difficult to envisage the potential of OV in the perioperative period following pancreatic resection, which often bears liver metastases and may have overlapping pathways of tissue repair and immunosuppression. On a clinical note, preventing immune suppression following surgery, could also have the added benefit of decreasing post-operative infections rates and this could lead to further improvements in survival for already fragile PDAC surgical patients.

## 8 Clinical considerations for perioperative virotherapy in PDAC patients

Currently, oncolytic virotherapy is experiencing a resurgence in research and clinical interest. However, it is a relatively young field of research. There is little human data and few clinical trials of its use in the perioperative period, let alone in PDAC. For future research, certain critical elements need to be prudently considered when planning clinical trials and future treatments.

Firstly, the timing of administration needs to be carefully evaluated. Matzner et al. make the compelling argument that the focus needs to be on the immediate perioperative period to start treatment (75). According to the National Cancer Institute, this perioperative period loosely refers to the weeks prior to surgery, followed by admission, hospitalization, preparations for surgery, anesthesia, and surgical procedures, as well as following convalescence and functional recovery. In clinical trials, interventions limited to the immediate perioperative period have been shown to improve disease-free survival and overall survival (12, 51, 120, 121). Furthermore, preclinical studies have demonstrated repeatedly the anti-metastatic effect of combining surgery with immunomodulatory approaches (51, 122, 123). Therefore, the scheduling of neoadjuvant virotherapy could be limited to this small treatment window before resection. Virotherapy could also be administered in the days following surgery to stay within the perioperative window. However, the immediate days following resection is a critical period for wound healing and tissue repair, especially in complex pancreas surgery (124). Any intervention that disrupts this complex organ and the physiological processes that it regulates, could result in catastrophic surgical complications such as post-operative pancreatic fistula or pancreatitis (7). The existing evidence appears to suggest that the immediate perioperative period is disproportionately critical in predicting long-term cancer outcomes. Therefore, any clinical success in this period yields the potential for long lasting effects on patients’ survival.

Secondly, we need to consider the best route of administration. As with any treatment, the administration method must be thoroughly investigated. This is critical when establishing an OV treatment for a disease as aggressive as PDAC. Most clinical trials of OVs in other solid tumors have disproportionately selected intratumoral administration to increase viral biodistribution to cancer cells and to prevent neutralization by systemic humoral immune responses. While the rationale is undeniable, it is important to understand the disease we are treating. The primary drawback of intratumoral delivery is the difficulty of reaching deep visceral tumors such as pancreatic cancer, particularly when repeated treatment is required (125). Therefore, when designing a novel OV trial for PDAC, this component needs to be factored in the decision-making process. Although endoscopic trans-gastric access permits intratumoral injection, repeated delivery may expose patients to an elevated risk of complications. In addition, endoscopic access may be difficult in the early post-operative period. Most patients who have Whipple surgery will have extensive restoration of their proximal digestive tract (3, 126). Therefore, endoscopic operations during the early perioperative period may significantly compromise the structural integrity of a newly created anastomosis and increase the chance of a catastrophic anastomotic leak. All things considered, intravenous administration of OV could be the more pragmatic and safer method for treating PDAC. There have been reports of successful intravenous administration of OV, but the therapeutic benefit has yet to be validated in larger, randomized clinical trials.

Finally, many other factors related to the OV under investigation should be taken into consideration. This includes choosing the appropriate viral backbone, arming, or not arming the virus with a payload, the type of payload to use, using OV as a monotherapy or in combination with existing standard-of-care chemotherapy or immunotherapy, etc. (125, 127). These are some virus-related factors to take into account for future PDAC trials, but a deeper discussion of these considerations goes beyond the scope of this review. One thing is certain, OV is an extremely versatile platform with great potential to both directly target pancreatic cancer and reverse the immunosuppressive effect of surgical stress on the pathophysiology of PDAC. Concerns related to the potential negative effects of virotherapy must be carefully weighed against the possible advantages of multi-pronged immunomodulation. As previously suggested, the immunostimulating capacity of OV might result in both improved survival outcomes and reduction in post-operative infectious complications. A delicate balance must be struck between immune activation and post-operative adverse events. However, the lack of perioperative clinical trials in surgical cancer patients do not allow us to directly assess these critical issues, leaving clinically relevant questions unanswered and underpinning mechanisms unknown.

## 9 Conclusions and perspectives

Due the improvements observed in the past decades for chemotherapeutic regimens (mFolfirinox, Gemcitabine-nab-Paclitaxel), better perioperative care and centralization of pancreatic surgery to high volume centers, neoadjuvant/perioperative immunotherapy for the treatment of PDAC has lost momentum (120). While mortality and morbidity following surgery have decreased (4, 128–130), survival, however, remains stagnant (2, 131). Emerging successes following immunotherapy treatments in other aggressive solid cancers provide the rationale to pursue immunotherapy and novel viro-immunotherapies in high-fatality PDAC. Given the cold immune phenotype of PDAC, we reason that oncolytic virotherapy, administered within the perioperative window of opportunity should be exploited to maximize survival benefits for patients. Oncolytic virotherapy, an emerging, multi-mechanistic therapy should be considered as viable therapeutics for the treatment of PDAC or as an essential component of multimodal therapy, which takes into consideration timing of treatment and benefits/drawbacks of frontline therapies. This will only be feasible with further research and clinical studies to understand the therapeutic potential of the perioperative period.

## Author contributions

SM and LHT conceived the main ideas for this paper, wrote and revised the manuscript. LD wrote and revised the manuscript. NA, ML, JEB, AAA and YC revised the manuscript. YC and LHT provided supervision for the activities of the manuscript. All authors contributed to the article and approved the submitted version.
